# Revolutionary hyaluronic acid-modified edge-activated spanlastics as a novel approach to boost Hepatoprotective activity of Curcumin: Optimization, biochemical analysis and *in-vivo* assessment

**DOI:** 10.1016/j.ijpx.2025.100430

**Published:** 2025-10-31

**Authors:** Sadek Ahmed, Osama Saher, Rana M. ElBishbishy, Mennatullah M. Ibrahim

**Affiliations:** a Department of Pharmaceutics and Industrial Pharmacy, Faculty of Pharmacy, Cairo University, Cairo, Egypt; b Department of Laboratory Medicine, Karolinska Institute, Stockholm, Sweden; Department of Cellular Therapy and Allogeneic Stem Cell Transplantation (CAST), Karolinska University Hospital Huddinge and Karolinska Comprehensive Cancer Center, Stockholm, Sweden; c Department of Pharmacology and Toxicology, Faculty of Pharmacy, Cairo University, Cairo, Egypt

**Keywords:** Curcumin, Spanlastics, Hyaluronic acid, Hepatoprotective, CCl₄-induced hepatotoxicity, Biochemical analysis

## Abstract

Drug-induced liver injury (DILI) represents a critical clinical problem that often necessitates lowering the therapeutic dose or even complete drug withdrawal, ultimately resulting in treatment failure. Curcumin (Cur), a natural polyphenolic compound, demonstrates strong hepatoprotective and antioxidant activity; however, its poor solubility and limited bioavailability hinder its therapeutic use. To overcome these limitations, the present study aimed to develop and optimize curcumin-loaded hyaluronic acid-modified edge-activated spanlastics (Cur-HES) as an efficient delivery system for enhancing the hepatoprotective efficacy of curcumin against carbon tetrachloride (CCl₄)-induced liver damage. Cur-HES were prepared using the ethanol injection method and systematically optimized *via* a 23 full factorial design, where the independent variables included hyaluronic acid-to-surfactant ratio (X1), edge activator-to-drug ratio (X2), and Span 80 % contribution (X3). Formulations were assessed for entrapment efficiency (EE%), particle size (PS), polydispersity index (PDI), and zeta potential (ZP). The optimized formulation achieved a desirability value of 0.982, with EE% of 88.4 %, PS of 105.2 nm, PDI of 0.19, and ZP of −20.9 mV. Transmission electron microscopy revealed spherical vesicles. *In-vitro* release exhibited biphasic Higuchi diffusion kinetics, while stability testing confirmed preservation of physicochemical properties for three months. *In-vivo* evaluation demonstrated that Cur-HES provided significantly greater hepatoprotection than free Cur in the CCl₄-induced hepatotoxicity model, as evidenced by marked reductions in serum ALT and AST levels. Histopathological examination supported these findings, showing preserved liver architecture in treated groups. Overall, Cur-HES represents a promising nanocarrier platform to boost the hepatoprotective activity of Cur, offering a safe and effective therapeutic strategy against DILI.

## Graphical abstract

**Figure:**
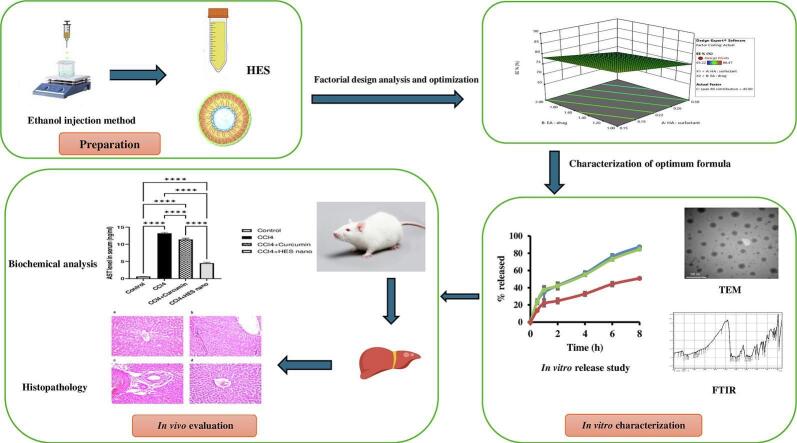

## 1 Introduction

As one of the body's most vital organs, the liver performs more than five hundred essential functions, including digestion, metabolism, and the removal of toxins (Kandimalla et al., 2016). Hepatotoxicity, or liver damage induced by exposure to toxic agents such as drugs, chemicals, or environmental pollutants, remains a serious clinical problem that can impair hepatic functions like bile secretion, storage, and metabolism (Wei et al., 2019). Among the key causes of acute liver failure is drug-induced liver injury (DILI), which also plays a major role in prompting drug recalls from the pharmaceutical market, creating a substantial economic burden (Li et al., 2022a). Hepatotoxicity is clinically manifested by symptoms such as fatigue, nausea, vomiting, abdominal pain, anorexia, jaundice, dark urine, and pale stool (Fahmy et al., 2025). Unfortunately, most treatment approaches remain symptomatic, mainly involving dose lowering or withdrawal of the offending drug, with limited effective therapeutic options available (Ruiz de Porras et al., 2023). Hence, there is an urgent need for more effective hepatoprotective strategies. In recent years, increasing research has addressed strategies to mitigate DILI, with particular attention to the use of antioxidants and naturally derived bioactive compounds, including flavonoids, polyphenols, carotenoids, and vitamins A and E (Liu et al., 2021; Wolf et al., 2008).

Among these, curcumin (Cur), the principal polyphenol found in turmeric (*Curcuma longa*), has been extensively studied (Khan et al., 2019). Curcumin exhibits multiple biological effects, including antioxidant, anti-inflammatory, antifibrotic, and hepatoprotective activities (Farzaei et al., 2018). Its hepatoprotective mechanisms involve quenching of free radicals, suppression of lipid peroxidation, suppression of pro-inflammatory mediators, and prevention of hepatic fibrosis through inhibition of HIF − 1α in an ERK-dependent manner (Fahmy et al., 2025; Khan et al., 2019). Despite these promising effects, curcumin's therapeutic efficacy is limited by poor aqueous solubility, instability in alkaline pH, rapid metabolism, and small systemic bioavailability (Tagde et al., 2021).

To overcome these drawbacks, curcumin has been incorporated into diverse nanocarrier systems that enhance its solubility, stability, and cellular uptake, thereby improving its bioavailability and therapeutic efficiency (Adel et al., 2021; Hande et al., 2019). Reported examples include Cur-loaded PLGA nanoparticles, polymeric micelles, phytosomes and cubosomes (Tagde et al., 2021; Choudhury et al., 2016). Such nanoformulations highlight the potential of advanced drug delivery systems in enhancing curcumin's hepatoprotective efficacy (Laurindo et al., 2023).

Carbon tetrachloride (CCl₄) stands among the most widely utilized hepatotoxins in preclinical studies, serving as a standard experimental model to investigate liver injury and assess the efficacy of hepatoprotective agents (Jia et al., 2014). This model is particularly valuable as it closely mimics the pathophysiology of human liver diseases, including fibrosis, cirrhosis, and acute liver failure (Jia et al., 2014). The hepatotoxic effect of CCl₄ arises from its metabolism by hepatic cytochrome P450 enzymes, which generates very reactive free radicals that trigger lipid peroxidation and subsequent hepatocellular damage (Dua et al., 2023; Christ et al., 2024). Moreover, CCl₄-induced hepatotoxicity is associated with angiogenesis, sinusoidal capillarization, and fibrosis, processes that Cur has been shown to counteract through suppression of multiple proangiogenic factors (Yao et al., 2013).

Nanotechnology has developed as a promising platform in drug delivery, offering innovative strategies to address the restrictions related to conventional oral delivery (Ibrahim et al., 2024a; Elmahboub et al., 2025). Many orally administered drugs suffer from poor aqueous solubility, significant first-pass metabolism, instability in the GIT, and consequently low systemic bioavailability, all of which reduce their therapeutic efficiency. Nanotechnology-based carriers can effectively address these challenges by enhancing solubility, protecting drugs from enzymatic degradation, prolonging gastrointestinal residence time, and promoting absorption across the intestinal mucosa (Faheem et al., 2025). Such advantages have expanded the application of nanocarrier systems in improving oral bioavailability and maximizing therapeutic outcomes.

Within this framework, spanlastics have gained considerable attention as flexible nanovesicular carriers mainly composed of nonionic surfactants like Span® combined with an edge activator (EA), which provides deformability to the vesicular membrane (Ansari et al., 2022a; El Hosary et al., 2024). In their structure, the bilayer-forming surfactants (*e.g.*, Span 80 and Tween 20) provide the vesicular framework, while the EA (such as Cremophor EL) destabilizes the bilayer, generating pores that enhance membrane elasticity and facilitate vesicular penetration through biological barriers (Al-mahallawi et al., 2017; Kakkar and Kaur, 2011). In comparison with conventional vesicles like liposomes or niosomes, which often suffer from rigidity and limited permeability, spanlastics exhibit superior flexibility and stability, allowing efficient encapsulation and delivery of both hydrophilic and hydrophobic drugs where these drugs are incorporated in the inside hydrophilic core and the outer lipid layer, respectively (Muzzalupo and Tavano, 2015). The inclusion of cholesterol in the formulation further contributes to structural integrity by stabilizing the lipid bilayer and preventing vesicle leakage (Kakkar and Kaur, 2011). Additionally, spanlastics are biodegradable, biocompatible, and exhibit low toxicity, which enhances their safety profile for clinical use (Younis et al., 2023). Due to their nanoscale size, high entrapment capability, high elasticity and enhanced stability, spanlastics have developed as a promising oral drug delivery system able to overcome gastrointestinal barriers and improving the solubility and bioavailability of poorly water-soluble drugs (Fatouh et al., 2022).

Spanlastics have been investigated across different administration routes, including topical, ocular, dermal, and oral delivery, with the latter showing remarkable potential in enhancing gastrointestinal absorption and bioavailability of poorly soluble drugs (Younis et al., 2023; Tayel et al., 2015; Kaur et al., 2012; Kakkar and Kaur, 2013). As a result of their structural flexibility and deformability, spanlastics can cross biological membranes efficiently, offering improved therapeutic efficiency (Benson, 2006). Such features highlight their potential for targeted applications, including the delivery of hepatoprotective agents like curcumin where liver-specific uptake and enhanced therapeutic outcomes are required.

Hyaluronic acid (HA) represents a natural glycosaminoglycan abundantly present in the extracellular matrix (ECM), where it plays a central role in maintaining hydration, viscoelasticity, and exhibits excellent biocompatibility (Ouyang et al., 2024). As a biodegradable, non-toxic, and non-immunogenic biopolymer, HA has been commonly incorporated in drug delivery systems to improve colloidal stability and promote mucoadhesion, thus facilitating gastrointestinal absorption upon oral administration (Fahmy et al., 2021; Yu et al., 2024; Saher et al., 2019). Of particular importance, HA exhibits a strong binding affinity to CD44 receptors, which are markedly overexpressed during liver inflammation, fibrosis, and hepatocellular carcinoma, thereby enabling selective hepatic uptake and providing an effective strategy for targeted drug delivery to diseased liver tissues (Li et al., 2020; Kim and Seki, 2023). HA-based targeting strategies have been successfully demonstrated in multiple studies. Yu et al. reported that HA-modified nanocarriers selectively accumulated in activated hepatic stellate cells *via* CD44 interactions, leading to superior antifibrotic activity (Yu et al., 2024). Moreover, HA has natural affinity for hepatic cells, particularly Kupffer cells, enables receptor-mediated uptake, which enhances cellular internalization of encapsulated agents, thereby boosting their hepatoprotective efficacy (Li et al., 2022b). Beyond its role as a targeting ligand, HA contributes indirectly to hepatoprotection because of its partial antioxidant properties, anti-inflammatory activity, and ability to promote tissue repair (Awadalla et al., 2023). Furthermore, HA may also contribute to improving the physicochemical performance of Cur. Its hydrophilic nature helps maintain the aqueous dispersibility of unentrapped Cur and reduces the risk of precipitation or crystallization (Manju and Sreenivasan, 2011). Therefore, integrating HA into elastic nanocarriers such as spanlastics provides not only enhanced stability and mucoadhesion of the vesicles but also receptor-mediated hepatic uptake, thereby maximizing the hepatoprotective potential of encapsulated agents.

Factorial experimental design provides a systematic and reliable tool for evaluating how formulation variables impact key quality characteristics, enabling the optimization of nanocarrier systems with high accuracy (Younes et al., 2024; Ahmed et al., 2025a). As far as we are aware, no previous reports have described the formulation of hyaluronic acid-modified edge-activated spanlastics (HES) for oral administration. In the present work, curcumin (Cur) was incorporated into HES, composed of Span 80, Tween 20, cholesterol, HA and Cremophor EL, using a full factorial design approach. The effect of formulation components and their ratios on particle size (PS), zeta potential (ZP), polydispersity index (PDI), and entrapment efficiency (EE %) was thoroughly investigated. The optimum Cur-HES formulation was further subjected to a series of *in vitro* characterizations, including transmission electron microscopy (TEM), FTIR, release profile, and physical stability studies. Moreover, *in vivo* evaluations were carried out in a CCl₄-induced hepatotoxicity model to assess hepatoprotective efficacy, uptake, and overall safety of the optimized nanocarrier. This strategy highlights the prospects of HES as a promising liver-targeted delivery system and a novel oral hepatoprotective nanocarrier for curcumin. Although the current study focused on preclinical evaluation, the developed Cur-HES system holds potential for future human oral application following further safety and pharmacokinetic investigations.

## 2 Materials and methodology

### 2.1 Materials

Curcumin (Cur, purity 95 %) was obtained from Fisher Scientific International, Inc. (Massachusetts, USA). Hyaluronic acid (HA), provided in the form of sodium hyaluronate with a molecular weight range of 400–800 kDa, was sourced from Acros Organics (Belgium). Cholesterol, Tween 20, and dialysis membranes with a molecular weight cutoff of approximately 12,000–14,000 Da were supplied by Sigma-Aldrich (St. Louis, MO, USA). Cremophor® EL (polyethoxylated castor oil) was purchased from BASF (Germany). Disodium hydrogen phosphate, potassium dihydrogen phosphate, ethanol (95 %), sodium chloride, carbon tetrachloride (CCl₄), and Span® 80 were kindly provided by El Nasr Pharmaceutical Chemicals Company (Cairo, Egypt). All other solvents and reagents employed in this study were of analytical grade and used directly without additional purification.

### 2.2 Experimental design

A 2^3^full factorial design was employed to study the impact of formulation variables on the characteristics of Cur-HES utilizing Design-Expert® software (version 12, Stat-Ease, Inc., Minneapolis, MN, USA). The 2^3^ factorial design was selected because its ease and efficacy (Ahmed et al., 2025g). The independent variables included the hyaluronic acid (HA)-to-surfactant (SAA) weight ratio (X_1_), the edge activator (EA)-to-drug (Cur) weight ratio (X_2_) and Span 80 contribution (% from the total SAA amount) (X_3_). The measured responses were entrapment efficiency (%EE, Y_1_), particle size (PS, Y_2_), polydispersity index (PDI, Y_3_), and zeta potential (ZP, Y_4_). The factors, their levels, and the corresponding responses with desirability constraints are presented in Table 1. The selected levels of the independent variables were determined based on preliminary exploratory trials that ensured the production of stable, homogeneous, and deformable vesicles suitable for further optimization.

**Table 1:** 2^3^ factorial design of Cur-HES formulations illustrating the selected factors, their levels, responses, and desirability constraints.

Factor (independent variable)	Level	
	-1	+1
X_1_: HA: SAA ratio	0.15	0.3
X_2_: EA**:** drug ratio	1	2
X_3_: Span 80 contribution (% of total SAA)	30	60
**Response (dependent variable)**	**Desirability constraints**	
Y1: EE %	Maximize	
Y2: PS (nm)	Minimize	
Y3: PDI	Minimize	
Y4: ZP (absolute value) (mV)	In Range	

Abbreviations: EE %, percent entrapment efficiency; Cur, curcumin; PDI, polydispersity index; PS, particle size; ZP, zeta potential; HA, hyaluronic acid; SAA, surfactant; EA; edge activator; HES, Hyaluronic Acid-Modified Edge-Activated Spanlastics.

### 2.3 Formulation of Cur-HES

Cur-HES were formulated *via* ethanol injection technique (El Hosary et al., 2024; Kakkar and Kaur, 2011). Briefly, curcumin (10 mg), cholesterol (30 mg), together with the specified amounts of Cremophor EL (edge activator) and surfactants (Span 80 and Tween 20, totaling 100 mg), were dissolved in ethanol while kept in a water bath at 60 °C. The resulting organic mixture was then gradually injected into an aqueous phase (two times the organic volume) under continuous stirring at the same temperature, till ethanol was fully evaporated, yielding a primary spanlastics dispersion. Hyaluronic acid was subsequently incorporated by sprinkling into the dispersion under stirring at room temperature until a uniform mixture was achieved. The resulting dispersion was subsequently sonicated for 5 min at 25 ± 2 °C (Ultra Sonicator, model LC 60/H, Elma, Singen, Germany) to reduce PS (Fahmy et al., 2025). The final Cur-HES dispersions were kept at 4 °C for further evaluation (Ibrahim et al., 2020; Daralnakhla et al., 2021). Table 2 summarizes the composition of the prepared Cur-HES formulae along with their evaluated responses.

**Table 2:** Composition and characterization of Cur-HES formulations prepared according to the 2^3^-factorial design, including independent variables and measured response.

Formulation	X_1_: HA: SAA ratio	X_2_: EA: drug ratio	X_3_: Span 80 %	Y_1_: EE %	Y_2_: PS (nm)	Y_3_: PDI	Y_4_: ZP (mV)
F1	0.15	1	60	74.5 ± 3.3	135.1 ± 0.2	0.14 ± 0.01	−26.4 ± 0.5
F2	0.3	2	30	79.6 ± 1.5	123.1 ± 0.9	0.18 ± 0.02	−33.3 ± 0.8
F3	0.15	2	30	86.2 ± 1.7	113.5 ± 0.9	0.14 ± 0.01	−21.8 ± 0.9
F4	0.15	1	30	88.4 ± 0.9	105.2 ± 1.6	0.19 ± 0.02	−20.9 ± 1.3
F5	0.3	2	60	65.2 ± 1.6	171.6 ± 1.7	0.31 ± 0.03	−43.9 ± 1.7
F6	0.3	1	30	82.5 ± 2.2	119.4 ± 0.7	0.15 ± 0.02	−29.7 ± 0.6
F7	0.3	1	60	67.4 ± 2.3	155.6 ± 1.6	0.24 ± 0.01	−34.7 ± 1.9
F8	0.15	2	60	70.2 ± 2.7	155.3 ± 3.1	0.26 ± 0.01	−26.6 ± 1.1

The data presented are the mean ± SD (*n* = 3).

Abbreviations: EE %, percent entrapment efficiency; Cur, curcumin; PDI, polydispersity index; PS, particle size; ZP, zeta potential; HA, hyaluronic acid; SAA, surfactant; EA; edge activator; HES, Hyaluronic Acid-Modified Edge-Activated Spanlastics.

### 2.4 *In-vitro* characterization of the formulated Cur-HES

#### 2.4.1 %EE determination

The percentage encapsulation efficiency of Cur was measured using the indirect method (Ahmed et al., 2025b; Ahmed et al., 2025c). In brief, an aliquot of the prepared Cur-HES dispersion (1 mL, correspondent to 0.5 mg Cur) was exposed to ultracentrifugation at 22,000 rpm for one hour at 4 °C by a cooling ultracentrifuge (3 K 30, Sigma, Germany). The amount of free (non-entrapped) Cur present in the supernatant was determined after appropriate dilution using a UV–visible spectrophotometer at 425 nm (λ_max_) (Shimadzu UV-1601 PC, Kyoto, Japan) (*n* = 3, R^2^ = 0.9995). The %EE was subsequently computed using the equation below (Ahmed et al., 2025d):

(1) $$\%\mathrm{EE}=\frac{\text{Total drug amount}-\text{free drug in the supernatant}}{\text{Total drug amount}}\times 100$$

#### 2.4.2 PS, PDI and ZP measurement

The PS, PDI, and ZP of the prepared Cur-HES formulae were determined by dynamic light scattering (DLS) with a Nano Zeta sizer (Malvern Instruments Ltd., Malvern, UK). Before measurements, the dispersions were properly diluted with distilled water (1:50) to obtain a translucent and homogenous suspension. Measurements were achieved at 25 °C in triplicate to ensure accuracy and reproducibility (Ahmed et al., 2025d). The PDI values were used as an indicator of the uniformity of vesicle size distribution, where lower values reflect narrower distribution and higher values indicate greater heterogeneity (Ibrahim et al., 2024b). ZP was determined using the same instrument equipped with a zeta potential cell, based on the electrophoretic movement of the charged vesicles in an applied electric field (Ahmed et al., 2025e). The mean ± SD values were calculated from three independent measurements for each formulation.

### 2.5 Statistical optimization of Cur-HES formulations

Numerical Optimization of the Cur-HES formulations was carried out utilizing Design-Expert® software. The process was directed by setting specific criteria for the investigated responses (%EE, PS, PDI, and ZP), with the goal of maximizing %EE and the absolute value of ZP, and minimizing PS and PDI, as detailed in Table 1. To assess the influence and statistical relevance of the formulation factors as well as their interactions on the studied responses, analysis of variance (ANOVA) was employed (Younes et al., 2024). The desirability function was then applied, where each response was first converted into a desirability index, and subsequently combined into an overall desirability score ranging from 0 (undesirable) to 1 (most desirable) (Ahmed et al., 2024a; Ahmed et al., 2022a). The Cur-HES formulation showing the greatest desirability value was chosen as the optimum formula and subjected to further evaluation. To ensure the accuracy of the optimization process, the optimal formulation was re-examined for %EE, PS, PDI, and ZP, and the observed values were statistically compared to the predicted ones. The deviation percentage between predicted and experimental results was determined using the following equation, where small deviation values indicate reliability of the optimization model (Ibrahim et al., 2024b; Ahmed et al., 2024a):

(2) $$\%\text{Deviation}=\frac{|\text{Predicted value}-\text{Observed value}|}{\text{Observed value}}\times 100\;$$

### 2.6 *In-vitro* evaluation of the optimal Cur-HES formulation

#### 2.6.1 Fourier-transform infrared spectroscopy (FTIR)

FTIR spectra were obtained for Cur, HA, cholesterol, and the lyophilized optimized Cur-HES formula using a Bruker FTIR spectrophotometer (model 22, Coventry, UK). This characterization was carried out to assess potential interactions between Cur and other formulation components in the prepared system (Ahmed et al., 2025c; Ahmed et al., 2024b). For each sample, an accurately weighed portion (3 mg) was thoroughly blended with dry KBr and pressed into a transparent pellet. The pellets were scanned at room temperature within the spectral range of 4000–500 cm^−1^ (Younes et al., 2024). The obtained spectra were then compared to detect characteristic peaks of the individual components and any potential shifts or alterations in the optimum formula, which may indicate drug–excipient interactions.

#### 2.6.2 Transmission electron microscopy (TEM)

The morphology of the optimized Cur-HES formulation was examined by Transmission Electron Microscopy (TEM, JEM-2100, JEOL, Japan). The sample was diluted with distilled water, applied to a carbon-coated copper grid, air-dried, and negatively stained with 1 % phosphotungstic acid to enhance contrast. The grids were then observed at 80 kV under suitable magnifications to visualize the vesicular structure (Younis et al., 2023; Ahmed et al., 2024c).

#### 2.6.3 Comparative *In-vitro* release study

The release behavior of the optimal Cur-HES formula was assessed using the dialysis bag diffusion method (Ahmed et al., 2024b). Briefly, dialysis membranes (Cutoff of 12,000–14,000 Da) were pre-soaked overnight in phosphate-buffered saline (PBS, pH 7.4). A quantity corresponding to 1 mg of Cur, either from the optimized Cur-HES or from an aqueous Cur suspension (control), was placed inside the hydrated dialysis bags and submerged in 50 mL PBS within amber glass containers (Sayed et al., 2024). The receptor medium was kept at 37 ± 0.5 °C in a shaking water bath (100 rpm) (Unimax, IKA, Staufen, Germany) to ensure uniform mixing. At predetermined time intervals (0.5, 1, 2, 4, 6, and 8 h), aliquots were withdrawn and immediately substituted with equal volume of fresh vehicle to conserve sink conditions (4). The samples underwent spectrophotometric analysis at λ_max_ 425 nm (*n* = 3, R^2^ = 0.9997) and the % cumulative drug release was plotted as a function of time. To elucidate the release mechanism, the obtained release results were applied to different kinetic models involving zero-order, first-order, Korsmeyer–Peppas and Higuchi diffusion models. The model providing the best fit was determined according to the highest correlation coefficient (R^2^) (Nemr et al., 2024; Tawfik et al., 2025).

#### 2.6.4 Antioxidant assay

The antioxidant potential of the optimized HES was evaluated using the 2,2-diphenyl-1-picrylhydrazyl (DPPH) radical scavenging assay. A freshly prepared methanolic DPPH solution (0.004 % *w*/*v*) was kept in the dark at 10 °C to maintain its stability prior to analysis. The test samples were dissolved in methanol, and 40 μL of each sample was mixed with 3 mL of the DPPH reagent. Immediately after mixing, the absorbance was recorded at 515 nm using a UV–visible spectrophotometer (Spectronic 1201, Milton Roy, USA). The decrease in absorbance, which reflects the free radical scavenging process, was monitored at one-minute intervals until a steady reading was achieved (Yen and Duh, 1994). To validate the procedure, ascorbic acid was employed as a reference antioxidant, while the DPPH reagent alone acted as the control. Each experiment was carried out in triplicate, and the average readings were used for analysis to ensure accuracy and reproducibility. The percentage of DPPH radical inhibition (PI) was determined using the following Eq. (Al Zahrani et al., 2020):

(3) $$\mathrm{PI}=\frac{\left(\mathrm{AC}-\mathrm{AT}\right)}{\mathrm{AC}}\;x\;100$$

Where AC is the initial absorbance of the control and AT is the absorbance of the test sample. Additionally, the half-maximal inhibitory concentration (IC₅₀) that represents the concentration of the sample required to neutralize 50 % of free radicals, was calculated. A lower IC₅₀ value indicated superior antioxidant efficiency (Yen and Duh, 1994).

#### 2.6.5 Physical stability study

To evaluate the stability of the optimal Cur-HES formulation, a short-term storage study was conducted. The formulation was kept in a tightly sealed container under refrigerated conditions (5 ± 3 °C) for three months (Fahmy et al., 2021). Following storage, the samples were visually inspected for any changes in physical appearance such as color, re-dispersibility, and presence of visible aggregates (Adel et al., 2021). In addition, the main physicochemical parameters including PS, PDI, % EE, and ZP were re-measured and compared to those of the freshly prepared formula. The values of PS, PDI, % EE, and ZP for the stored formulation were compared with those of the fresh preparation using one-way ANOVA. Moreover, the release profiles of fresh and stored samples were compared by calculating the similarity factor (ƒ2) using the equation below (Ahmed et al., 2025f):

(4) $$f_{2}=50.\log\left\{\;{\left[1+\left(\frac{1}{n}\right)\;\sum_{t=1}^{n}{\left(\mathrm{Rt}-\mathrm{Tt}\right)}^{2}\right]}^{-0.5}\times 100\right\}$$

Where *n* denotes the number of sampling points, and *Rₜ* and *Tₜ* correspond to the cumulative % of drug released at time *t* from the fresh and stored formulations, respectively. A value ranging between 50 and 100 reflects closeness between the two release profiles and indicates similarity in release behavior after storage (Ibrahim et al., 2024b).

### 2.7 *In vivo* study of the optimized Cur-HES (biochemical analysis)

#### 2.7.1 Animals

Adult male Wistar rats (with a body weight of approximately 250 g, aged 12 weeks) were engaged in the study. Rats were obtained from the colony of animals of the NODCAR (Giza, Egypt). Environmental conditions were kept optimum, and rats were held in a soundproof, temperature-regulated (25 ± 2 °C), humidity-controlled (60 ± 10 %) animal facility, as well as alternating light/dark cycle (12 h each). Furthermore, animals were permitted unlimited access to water and a regular chow feed.

The study obeyed the standards delineated in the Guide for the Care and Use of Laboratory Animals, in line with the US National Institutes of Health (Publication No. 85–23, revised 2011). Research Ethics Committee for Experimental and Clinical Studies at the Faculty of Pharmacy, Cairo University (REC-FOPCU), Cairo, Egypt, permitted the experiment (Approval No. PI 3677). Animals' well-being was taken as a priority in order to minimize any suffering throughout the research. The study was conducted in full compliance with the ARRIVE guidelines, guaranteeing precise reporting and safeguarding animal welfare.

#### 2.7.2 General experimental procedures

Rats were allocated at random into four groups (*n* = 6/group) and were treated as follows. Group I, which acted as negative control, rats received an oral daily dose of 0.2 mL saline. Group II, III, and IV received CCl4 twice weekly (0.5 mL/kg; dissolved in olive oil in 1:3 ratio) for one month (Mansour et al., 2022). Group II served as the insult group, while group III was treated daily with free Cur (25 mg/kg; p.o), solubilized in normal saline with 100 μL of Tween 80, to function as the Cur-treated group (Marslin et al., 2018). In parallel, group IV rats were daily treated with a freshly prepared optimized Cur-HES formulation (25 mg/kg; p.o), serving as the nano-formula-treated group. In this study, Tween 80 was included solely to enhance the aqueous solubility of curcumin, ensuring uniform dispersion and accurate dosing. The control group received saline only, as Tween 80 was not required in the absence of curcumin. It is important to note that Tween 80 at low concentrations has been widely reported in preclinical studies to exhibit minimal cytotoxicity (Kaur and Mehta, 2017).

#### 2.7.3 Samples collection

At the end of the treatment regimen, rats were anaesthetized by ketamine (50 mg/kg; i.p) and xylazine (10 mg/kg; i.p) (Green et al., 2016) to collect the blood samples. Blood was collected from eye-canthus in Wasserman tubes and kept in inclined position till time of centrifugation at a temperature of 4 °C, rotated at 3000 rpm for a period of 15 min. Following the centrifugation, serum was separated as transparent, non-hemolyzed supernatant and kept at −20 °C till biochemically analyzed. Afterwards, rats were euthanized by cervical dislocation and their liver was rapidly removed and sliced into two parts. The first part was fixed in 10 % (*v*/v) formalin till histological investigation. Inline, the second part was homogenized in phosphate-buffered saline to prepare 10 % (*w*/*v*) homogenate. The produced homogenates were separated into multiple aliquots and retained at −80 °C until subsequent testing.

#### 2.7.4 Biochemical parameters

##### 2.7.4.1 Liver function tests

AST level in serum was assessed by colorimetric assay utilizing Rat AST ELISA Kit (cat#: *E*-EL-R0076). On the other hand, ALT activity in serum was examined *via* ALT Activity Assay Kit (cat#: E-BC-K235-M). Both kits were obtained from Elabscience (TX, USA). The assay was done according to the procedures provided by the manufacturer.

##### 2.7.4.2 Inflammatory marker

TNF-α levels were examined by ELISA kit purchased from Elabscience (TX, USA, cat#: E-EL-R2856). Methods were done in accordance with the guidelines given by the manufacturer. Obtained results were reported as pg/mg protein. Lowry method, a method used to assay the protein content in tissue homogenate, was utilized (Lowry et al., 1951).

#### 2.7.5 Histological examination

To assess the general morphology of the liver, H&E dye was used. The tissue was fixed with phosphate buffered formalin for 72 h. Fixed tissue was dehydrated with a serial grade of alcohol. Dehydrated tissue was mounted in paraffin and cut into sections with a thickness of 3 μm. It can be stored at room temperature in light proof and moisture proof containers. Afterwards, xylene was used to dewax the paraffin-embedded slices and rinsed with hematoxylin eosin gradient ethanol for five minutes. Once dehydrated with conventional ethanol, slices were sealed and examined.

#### 2.7.6 Statistical analysis

Shapiro–Wilk test and Brown–Forsythe test were used to ensure data's normality and homogeneity respectively. For analyzing parametric data, one-way ANOVA was implemented, which was then followed by Tukey *post hoc* test. Data was displayed as mean ± standard deviation (SD). GraphPad Prism software (Version 9, San Diego, California, USA) was used for statistical analysis. *P*-value was kept below 0.05 as a threshold for significance for all comparisons.

## 3 Results and discussions

Cur-HES were formulated *via* ethanol injection method through a 2^3^ factorial design, generating eight formulations. Each formula was evaluated in terms of %EE, PS, PDI, and ZP to identify the optimal system for subsequent *in vitro* characterization and *in vivo* assessment. The detailed compositions and measured responses of all prepared formulations are shown in Table 2.

### 3.1 Analysis of factorial design

Factorial design represents a powerful statistical tool to explore the impact of formulation variables on the characteristics of drug delivery systems. In the present work, a 2^3^ full factorial design was utilized due to its efficacy in formulating and characterizing HES dispersions. Each measured response was fitted to the most suitable polynomial model, and model adequacy was verified using multiple statistical parameters. In particular, the agreement between adjusted and predicted R^2^ values (difference < 0.2) confirmed the predictive accuracy of the model (Ahmed et al., 2024a). Moreover, the adequate precision values were higher than 4, confirming that the model can reliably predict the responses within the design space (Ahmed et al., 2025a). As summarized in Table 3, these outcomes validate the model's capability for predicting the main responses (Y_1_: % EE, Y_2_: PS, Y_3_: PDI and Y_4_: ZP) and thus support its suitability for optimization purposes.

**Table 3:** Regression analysis for the investigated responses of Cur-HES.

Response	R^2^	Adjusted R^2^	Predicted R^2^	Adequate precision	Significant factors	Regression equation for each response
Y_1_: EE %	0.9973	0.9953	0.9892	56.440	X_1_, X_2_, X_3_	EE % = 76.75–3.09x_1_–1.47x_2_–7.41x_3_
Y_2_: PS (nm)	0.9733	0.9532	0.8930	18.339	X_1_, X_2_, X_3_	PS = 134.84 + 7.58x_1_ + 6.03x_2_ + 19.54x_3_
Y_4_: ZP (mV)	0.9282	0.8743	0.7127	11.301	X_1_, X_3_	ZP = 29.65 + 5.72x_1_+ 3.21x_3_

Abbreviations: EE %, percent entrapment efficiency; Cur, curcumin; PS, particle size; ZP, zeta potential; PDI, polydispersity index; HES, Hyaluronic Acid-Modified Edge-Activated Spanlastics.

The model of Y_3_ (PDI) was statistically insignificant (*p* > 0.05) and excluded from optimization.

#### 3.1.1 Model analysis of % EE

**Fig. 1:**
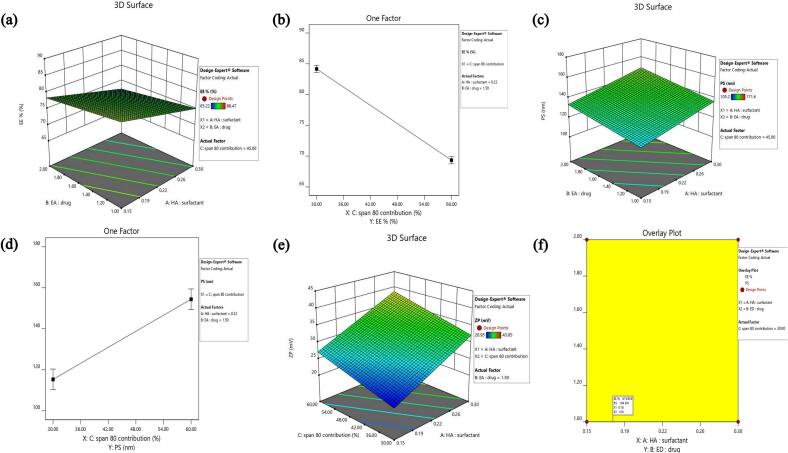
3D response surface plot for the effect of factor X_1_, (HA: surfactant ratio) and factor X_2_, (EA: drug ratio) on EE% (a), with line plot for the effect of factor X_3_, (Span 80 contribution %) on EE% (b), 3D response surface plot for the effect of factor X_1_, (HA: surfactant ratio) and factor X_2_, (EA: drug ratio) on PS (c), with line plot for the effect of factor X_3_, (Span 80 contribution %) on PS (d), 3D response surface plot for the effect of factor X_1_, (HA: surfactant ratio) and factor X_3_, (Span 80 contribution %) on ZP (e) and overlay plot illustrating the optimal design space corresponding to the highest desirability region (f).

Achieving high %EE is a significant requirement for oral delivery systems, particularly when the therapeutic objective is to improve the hepatoprotective potential of poorly soluble agents such as Cur (Fahmy et al., 2025). The %EE of the prepared Cur-HES formulae ranged between 65.2 ± 1.6 % and 88.4 ± 0.9 % **(**Table 2**)**, reflecting the efficiency of the system in entrapping Cur within the vesicular structure. ANOVA analysis confirmed the statistical significance of the applied model (*p* = 0.0001), supporting the reliability of the observed responses (statistical significance was determined at *p* < 0.05). A summary of regression coefficients and related significance values is provided in Table 3, while the impacts of the independent factors on %EE (Y_1_) are graphically shown in Fig. 1 **(a& b)**. Notably, all three independent factors, HA:SAA ratio (X_1_), EA: drug ratio (X_2_), and Span 80 % contribution (X_3_), showed significant negative effects (p < 0.05) on %EE. The possible explanations for these observations are discussed below.

Regarding factor X_1_ (HA: SAA ratio), increasing the proportion of HA was found to exert a significant negative effect on the %EE of Cur in the developed Cur-HES formulations. This reduction may be caused by the partial destabilization and disruption of the vesicular bilayer caused by the incorporation of higher amounts of HA, which promotes drug leakage into the external medium (Fahmy et al., 2021). Furthermore, the strong hydrophilicity and relatively high viscosity of HA solutions enhance the diffusion of Cur into the external phase, thereby lowering the fraction entrapped within vesicles. Moreover, the negative influence of HA on %EE can also be explained by its ability to increase Cur solubility in the aqueous environment, favoring its migration outside the vesicles (Cannito et al., 2022). The bulky polymeric chains of HA may further hinder the tight closure of the bilayer during vesicle formation and can interact with Cur molecules *via* hydrogen bonding or electrostatic forces, drawing them towards the outer medium instead of remaining entrapped (Tran et al., 2014). In addition, the possible formation of an external HA coating around the vesicles could alter the distribution of Cur between internal and external compartments, thus increasing the proportion of free, unentrapped drug (Khalid et al., 2023). Comparable results were reported by Wadhwa et al., who demonstrated a marked decrease in %EE upon increasing the concentration of sodium hyaluronate in chitosan-based nanoparticles (Wadhwa et al., 2010). Fahmy et al. also observed an inverse relation between HA concentration and the %EE in formulation of voriconazole-loaded HA enriched ultradeformable elastosomes for ocular delivery (Fahmy et al., 2021).

Considering factor X_2_ (EA: drug ratio), the EE% of Cur in the developed HES was observed to decrease significantly with increasing concentrations of the EA (Cremophor EL). This negative impact can be attributed to the membrane-destabilizing effect exerted by high levels of EA. Specifically, elevated Cremophor EL concentrations can induce pore formation within the vesicular bilayer, thereby disrupting membrane integrity and facilitating leakage of encapsulated Cur (Fahmy et al., 2021; Duangjit et al., 2011; Salama et al., 2012). Furthermore, when Cremophor EL levels reach its critical micelle concentration (CMC), micelle formation may occur, leading to partial solubilization of the drug in the aqueous phase rather than its entrapment within the vesicles, resulting in further reduction of EE% (Fatouh et al., 2022; Uchegbu and Florence, 1995). In agreement, Al-Mahallawi et al. reported that increasing EA concentrations adversely affected drug entrapment in transfersomal formulations (Al-mahallawi et al., 2014). Another explanation is related to the hydrophilic nature of Cremophor EL (HLB ≈ 12–14), which enhances vesicular hydration and imposes steric hindrance, reducing bilayer compactness and allowing Cur diffusion to the external medium (González-Rodríguez et al., 2007). The bulky molecular structure of Cremophor EL can also hinder proper bilayer closure and disturb vesicle packing, thereby promoting drug leakage (Al-mahallawi et al., 2017; Ahmed et al., 2025b). Additionally, high EA concentrations can increase vesicular membrane fluidity, which further compromises drug retention and facilitates leakage of entrapped molecules (Duangjit et al., 2011; Gupta et al., 2005). Similar results have been documented in the literature, where higher concentrations of edge activators were shown to decrease EE% due to enhanced bilayer permeability and pore formation, leading to drug loss into the external aqueous medium (Fatouh et al., 2022; Ahmed et al., 2025b; Salama et al., 2012; Al-mahallawi et al., 2014).

For factor X_3_ (Span 80 % contribution relative to total SAA), Increasing the proportion of Span 80 relative to Tween 20 significantly decreased the EE% of Cur-HES. This outcome appears contrary to expectations. Although Span 80 possesses favorable characteristics for drug entrapment, including a larger alkyl chain compared to Tween 20 and a lower hydrophilic-lipophilic balance (HLB ≈ 4.3), which generally support higher EE% through enhanced hydrophobic interactions within the vesicular bilayer (Ahmed et al., 2025a), a reduction in the overall EE% was still observed with increasing proportion of Span 80. This reduction is primarily attributed to the unsaturated alkyl chain containing a double bond in Span 80, which introduces bends in the hydrocarbon chain and prevents tight packing, thereby increasing membrane fluidity and permeability and facilitating Cur leakage (Abdelbary and El-Gendy, 2008). Additionally, Span 80 has a relatively low phase transition temperature (Tc) compared to saturated surfactants, which diminishes bilayer rigidity and further weakens vesicle stability, allowing more drug to escape during preparation (Nematollahi et al., 2017; Dharashivkar et al., 2014). By contrast, Tween 20, with its saturated alkyl chain and higher HLB (HLB ≈ 16.7), promotes tighter bilayer organization and stronger drug retention. Therefore, although Span 80 provides high lipophilicity and long chain length, the combined effects of chain unsaturation and low Tc override these advantages, ultimately lowering EE%. Similar findings have been reported in earlier studies, where vesicles prepared with unsaturated surfactants such as Span 80 or Tween 80 exhibited lower entrapment efficiency than those prepared with saturated counterparts like Span 60 or Tween 60, due to the increased bilayer permeability and reduced membrane compactness induced by unsaturation (Mazyed et al., 2021; Abou-Taleb et al., 2018). Taken together, these findings highlight that all studied formulation variables exerted a significant negative effect on entrapment efficiency, underscoring the critical need to optimize HA concentration, EA concentration, and Span 80 proportion to achieve stable vesicles with satisfactory drug retention.

#### 3.1.2 Model analysis of PS and PDI

The PS of the formulated Cur-HES ranged between 105.2 ± 1.6 and 171.6 ± 1.7 nm, as detailed in Table 2. This nanometer-scale size indicates that all prepared vesicles were within the optimal range for enhanced oral absorption and intracellular delivery. The size of the vesicles, influenced by formulation components along with their surface charge, significantly affects the internalization and accumulation of Cur within hepatocytes (Kusumoputro et al., 2023). ANOVA analysis confirmed the statistical significance of the model (*p* = 0.0013), with Table 3 presenting the regression results for PS (Y_2_) alongside the coded equation linking PS to the independent variables. ANOVA analysis revealed that all three independent variables, HA-to-SAA ratio (X_1_), EA-to-drug ratio (X_2_), and Span 80 contribution (% of total SAA, X_3_), significantly affected PS of Cur-HES (*p* < 0.05), as summarized in Table 3. The response surface plot (Fig. 1c) and the line plot **(**Fig. 1d) illustrate the effects of the significant factors on PS.

Regarding the effect of factor X_1,_ there is a positive correlation between HA content and PS which can be attributed to several factors. HA chains adsorb onto the vesicular surface *via* hydrogen bonding and hydrophilic interactions with surfactants, forming a hydrated coating layer that enlarges the hydrodynamic diameter as HA concentration increases (Ahmed et al., 2025g, Fahmy et al., 2021). The strong affinity of HA for water molecules further promotes the formation of a thick hydration shell around the vesicles, contributing to the apparent size increase (Sadeghi-Ghadi et al., 2021). Additionally, the intrinsic viscosity of HA can hinder efficient vesicle subdivision during preparation, favoring larger vesicle formation. Moreover, the negative charges introduced by HA also increase electrostatic repulsion between vesicles, while steric hindrance from its long polymeric chains contributes to apparent vesicle enlargement (Khalid et al., 2023). These observations agree with findings observed by Tran et al., Wadhwa et al. and Fahmy et al., who documented increased particle sizes with higher HA concentrations in different nanocarrier systems (Fahmy et al., 2021; Tran et al., 2014; Wadhwa et al., 2010).

Factor X_2_ (EA-to-drug ratio) also positively affected PS. Increasing the proportion of EA (Cremophor EL) from 1 to 2 resulted in the formation of larger vesicles. Hydrophilic surfactants like Cremophor EL (HLB ≈ 12–14) generally form larger vesicles due to their high surface free energy (Abdelkader et al., 2010). Increasing in PS can also be caused by the bulky hydrophilic head groups of Cremophor EL, which produce extensive hydration layers and steric stabilization around the vesicles, thereby reducing bilayer packing tightness and promoting vesicle enlargement (Ahmed et al., 2025b). Similar findings were reported by El Zaafarany et al., who reported vesicle enlargement with increasing EA concentration until a threshold, after which size reduction was noted (El Zaafarany et al., 2010). Ahmed et al. also observed a direct relation between EA concentration and the particle size of Terconazole loaded edge-activated hybrid elastosome (Ahmed et al., 2025b).

Similarly, Span 80 contribution (% of total SAA) (factor X_3_) also positively influenced PS. An increase in the proportion of Span 80 (HLB ≈ 4.3) relative to Tween 20 (HLB ≈ 16.7) resulted in larger vesicles. This effect is primarily due to the highly hydrophobic nature of Span 80 and its long alkyl chains, which enhance the critical packing parameter, promoting vesicle enlargement (El Hosary et al., 2024; Younis et al., 2023; Uchegbu and Florence, 1995). Moreover, increasing the proportion of Span 80 affects the vesicular membrane architecture, disrupting packing compactness, enhancing membrane flexibility and bending, and thereby promoting the generation of larger vesicles (Ahmed et al., 2025c; Elsherif et al., 2017). Similar outcomes have been noted in the literature by Aziz et al. (Aziz et al., 2022), Nemr et al. (Nemr et al., 2021), and Ahmed et al. (Ahmed et al., 2025b), confirming the role of hydrophobic surfactants in enlarging vesicle size.

The PDI serves as a key parameter to evaluate the uniformity of particle size distribution within vesicular formulations, reflecting both homogeneity and reproducibility of the preparation method (Nemr et al., 2021; Clayton et al., 2016). PDI values range from 0 to 1, with smaller values indicating higher uniformity; typically, values below 0.5 are indicative of a narrow size distribution (ElMeshad and Mohsen, 2016). In the present study, the PDI of the developed Cur-HES ranged between 0.14 ± 0.01 and 0.31 ± 0.03 (Table 2), suggesting a highly uniform particle distribution. Statistical analysis using ANOVA confirmed that the PDI model (Y_3_) was statistically insignificant (*p* = 0.239) and none of the independent factors had a significant effect on PDI (*p* > 0.05). Consequently, PDI was not included as a criterion in the numerical optimization process for selecting the optimized Cur-HES formulation.

#### 3.1.3 Model analysis of ZP

ZP is a crucial parameter reflecting the surface charge of vesicles and directly influences colloidal stability through electrostatic repulsion between particles (Ahmed et al., 2024a; Dubey et al., 2018). Higher absolute ZP values are associated with stronger repulsive forces, which minimize vesicle aggregation and improve the stability of nanosystems (Teama et al., 2025). Typically, vesicles with ZP magnitudes higher than ±20 mV are regarded as stable due to sufficient repulsive interactions preventing flocculation or fusion (Ahmed et al., 2025c). In the current study, ZP values of the prepared Cur-HES formulations ranged from −20.9 ± 1.3 to – 43.9 ± 1.7 mV **(**Table 2). These values indicate that all formulations possess acceptable stability. The negative surface charge can be ascribed to the presence of hydroxyl groups in Cur and the carboxyl functionalities of HA, in addition to the ionizable groups of the employed surfactants, which collectively confer a negative charge to the vesicular surface (Fahmy et al., 2025; Fahmy et al., 2021). For clarity in interpretation, statistical analysis of ZP results was carried out using absolute values. ANOVA analysis confirmed the statistical significance of the model (*p* = 0.0094). The regression results for ZP (Y_4_), together with the coded mathematical model describing its relationship with the studied independent variables, are presented in Table 3. Among the studied factors, X_1_ (HA: SAA ratio) and X_3_ (Span 80 % contribution relative to total SAA) exerted significant effects on ZP (*P* > 0.05), as illustrated in the response surface plots **(**Fig. 1e**).**

Regarding factor X_1_ (HA: SAA ratio), the increase in HA proportion exerted a positive effect on the magnitude of ZP, as higher HA concentrations enhanced the negative surface charge of Cur-HES vesicles. This can be explained by the presence of ionizable carboxyl groups in HA chains, which are adsorbed onto the vesicle surface and remain deprotonated at physiological pH, thereby increasing the net negative charge density and strengthening electrostatic repulsion among vesicles (Fahmy et al., 2021; Khalid et al., 2023). Such an effect ultimately improves colloidal stability and minimizes the risk of aggregation. Moreover, the external HA layer provides steric hindrance, which helps maintain dispersion uniformity and colloidal stability. Comparable findings were informed by Fahmy et al. and Wadhwa et al. (Fahmy et al., 2021; Wadhwa et al., 2010).

For factor X_3_ (Span 80 % contribution relative to total SAA), increasing the proportion of Span 80 relative to Tween 20 exerted a positive effect on the magnitude of zeta potential, as formulations showed more negative surface charges with higher Span 80 content. This effect can be explained by the greater incorporation of Span 80 molecules into the vesicular bilayer, which increases the number of hydroxyl groups available on the vesicle surface. These groups favor the selective adsorption of hydroxyl ions from the aqueous medium, thereby amplifying the net negative charge density (Ahmed et al., 2025a; Ahmed et al., 2024c). Moreover, Span 80 is more lipophilic (HLB ≈ 4.3) than Tween 20 (HLB ≈ 16.7), and this shift towards a more lipophilic surfactant composition reduces the masking of surface charges by hydrophilic moieties, allowing a stronger expression of negative charges at the interface (Aziz et al., 2018). Additionally, the unsaturated alkyl chain of Span 80, containing a double bond, introduces kinks that disturb bilayer packing and expose polar groups, further intensifying the negative ZP (Ahmed et al., 2025b). The predominance of Span 80 also facilitates stronger adsorption of HA chains onto the vesicle surface, contributing additional carboxylate groups and reinforcing electrostatic repulsion between vesicles. Importantly, higher ZP values play a critical role in colloidal stability, as the stronger electrostatic repulsion between particles minimizes the risk of aggregation and ensures uniform dispersion during storage (Ahmed et al., 2025a). Similar findings were reported in previous studies, where formulations enriched with lipophilic and unsaturated surfactants such as Span 80 exhibited higher negative ZP values, thereby improving their stability (Fatouh et al., 2022; Ahmed et al., 2025b).

### 3.2 Statistical optimization of Cur-HES

Design-Expert software was employed to identify the optimum Cur-HES formulation using numerical optimization techniques. The objective of the optimization process was to reveal the most suitable factor levels that yield the best vesicular characteristics. Based on the experimental results EE%, PS, and ZP, the software generated several candidate formulations. However, the PDI model (Y_3_) was statistically insignificant (*p* > 0.05), since all formulations exhibited acceptable homogeneity, and was thus excluded from the optimization process. For the optimization criteria, EE% was set to be maximized to ensure higher drug loading, while PS was targeted to be minimized in order to achieve smaller vesicles favorable for oral delivery. ZP, on the other hand, was maintained within a defined range rather than maximized, since all formulations already exhibited sufficiently high negative values (> ±20 mV), ensuring adequate colloidal stability. Selecting ZP “in range” rather than maximizing it was necessary to avoid choosing formulations with excessively high ZP values that were associated with undesirably larger PS and lower EE%. In fact, the consistently high negative ZP values across all formulations confirmed their stability, as values exceeding ±20 mV are generally sufficient to provide strong electrostatic repulsion and prevent vesicle aggregation, irrespective of whether the exact magnitude is higher or lower within this range (Ibrahim et al., 2024b). For each suggested Cur-HES formulation, a desirability value between 0 and 1 was generated according to the outcomes of %EE, PS, and ZP. Using numerical optimization, the software proposed an optimum composition that satisfied the selected criteria, achieving a high desirability score of 0.982. The suggested composition of the optimized formula was determined to be a HA-to-SAA ratio (X_1_) of 0.15, EA-to-drug ratio (X_2_) of 1, and a Span 80 % (X_3_) of 30 %, achieving a desirability score of 0.982. An overlay plot was constructed using Design Expert software to visualize the design space and the optimal region corresponding to the highest desirability value **(**Fig. 1f).

To validate this optimization, the predicted optimum Cur-HES formulation was actually prepared and characterized under the same conditions as the previously developed formulations. The observed data for %EE, PS, and ZP were then compared to the predicted values, and the residuals representing the differences between observed and predicted responses were calculated (Table 4). All responses exhibited very small residual values, not exceeding 0.9, which confirms the reliability and predictive power of the model. Moreover, the calculated percentage deviation between experimental and predicted results for all dependent variables remained below 5 % (Table 4), further supporting the accuracy of the optimization outcomes. These results, together with the strong consistency between adjusted and predicted R^2^ values in the regression analysis, demonstrated the robustness and validity of the final models. Consequently, the optimized Cur-HES formulation was selected for subsequent *in vitro* and *in vivo* investigations.

**Table 4:** Optimized Cur-HES formulation showing the selected levels of the independent variables along with the predicted and observed response values, as well as residual values.

Factor	Optimum level			
X_1_: HA**:** SAA ratio	0.15			
X_2_: EA**:** drug ratio	1			
X_3_: Span 80 (% of total SAA)	30			
Response	Predicted value	Observed value	Residual value a	% deviation
Y_1_: EE %	87.7	88.4	−0.79	0.90
Y_2_: PS (nm)	104.3	105.2	−0.90	0.86
Y_4_: ZP (mV)	−20.7	−20.9	0.2	1.19

For the % deviation, regard it as an absolute value.

Abbreviations: EE %, percent entrapment efficiency; Cur, curcumin; PS, particle size; ZP, zeta potential; HA, hyaluronic acid; SAA, surfactant; EA; edge activator; HES, Hyaluronic Acid-Modified Edge-Activated Spanlastics.

a Residual value = predicted value-observed value (absolute value).

### 3.3 *In vitro* characterization of the optimized Cur-HES

#### 3.3.1 FTIR

**Fig. 2:**
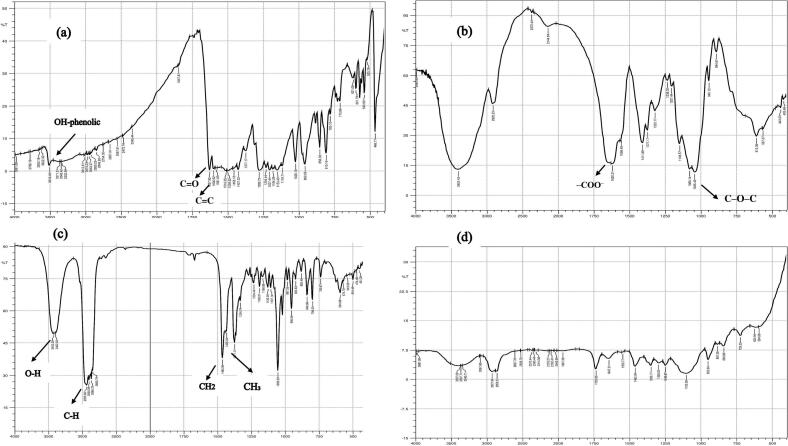
FTIR spectra of pure Cur (a), HA (b), cholesterol (c) and lyophilized Optimized Cur-HES formula (d). Arrows are used to indicate the characteristic absorption peaks of the functional groups.

The FTIR spectra of pure Cur, HA, cholesterol, and lyophilized optimal Cur-HES formula are presented in Fig. 2 and were investigated to confirm the successful encapsulation of Cur within the developed system. Pure Cur exhibited its characteristic absorption bands, including a broad peak around 3510–3515 cm^−1^ related to the stretching vibration of phenolic –OH groups. Distinct bands at 1625–1630 cm^−1^ and 1600 cm^−1^ were corresponding to C=O and C=C stretching vibrations, respectively, while aromatic C=C stretching appeared at 1500–1505 cm^−**1**^, consistent with previously reported data (Adel et al., 2021). The FTIR spectrum of HA displayed a broad band near 3250–3260 cm^−1^, attributed to O—H stretching, and a peak at 1600–1610 cm^−1^, related to the asymmetric stretching of the carboxylate (–COO^−^) groups. Additionally, a band around 1040–1050 cm^−1^ has been reported in the literature, representing C–O–C stretching of the glycosidic linkages in HA, further confirming its structure (Li et al., 2020; Manju and Sreenivasan, 2011). Cholesterol showed characteristic absorptions at 3415–3420 cm^−1^ for O—H stretching and at 2800–3000 cm^−1^ for aliphatic C—H stretching. Bending vibrations were detected at 1460–1470 cm^−1^ (CH₂ scissoring) and 1375–1380 cm^−1^ (CH₃ symmetric bending), while the fingerprint region exhibited peaks between 1050 and 1150 cm^−1^, assigned to C—O stretching of the hydroxyl group. These peaks (Romano et al., 2024) confirm cholesterol's molecular structure. The FTIR spectrum of the optimum Cur-HES formula revealed the disappearance of the characteristic Cur bands. The absence of distinct Cur peaks within the Cur-HES spectrum indicates its successful entrapment within the vesicular spanlastics dispersion. This confirms the molecular encapsulation of Cur in the developed nanocarrier system (Ahmed et al., 2022b; Kaur et al., 2022).

#### 3.3.2 Tem

**Fig. 3:**
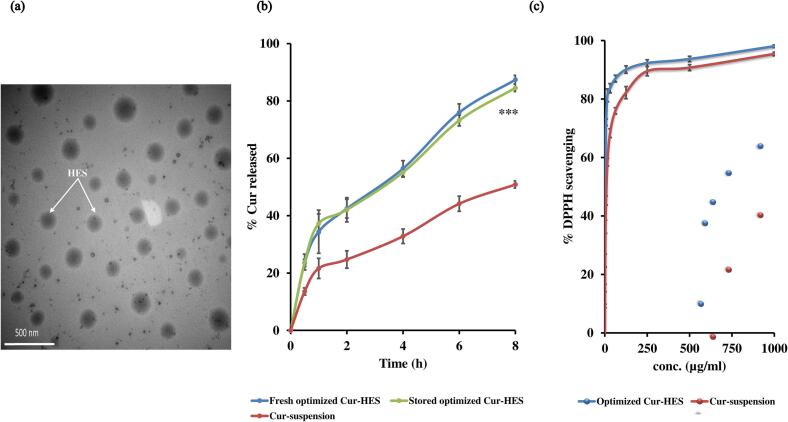
TEM micrograph (a) *In vitro* release profiles o (b) and *In-vitro* antioxidant activity (DPPH analysis) (c) of the optimized Cur-HES formula. Results are presented as mean ± SD, *n* = 3. *** indicate a highly significant difference (*p* < 0.001) between the Cur-HES formulations and the Cur suspension.

TEM examination was performed to examine the morphological characters and confirm the nanoscale architecture of the optimized Cur-HES formulation. The obtained images **(**Fig. 3a**)** revealed clearly defined, spherical vesicles with smooth surfaces and uniform size distribution. Importantly, the vesicles exhibited no evidence of aggregation, which highlights the structural stability of the system. This stability is likely due to the effects of the surfactants used in the formulation. The particle sizes observed under TEM were consistent with those obtained by DLS, demonstrating the reliability of the applied preparation technique and the validity of the size measurements (Aziz et al., 2023; Sakr et al., 2023).

#### 3.3.3 *In vitro* release study

In order to achieve an efficient release profile that guarantees therapeutic efficacy, it is essential to maintain a proper balance between hydrophilic components, which facilitate drug dissolution and diffusion, and hydrophobic ones, which sustain drug retention and prolong release (Ammar et al., 2020). The release pattern of Cur from the optimized Cur-HES formulation in PBS (pH 7.4) displayed a biphasic behavior (Fig. 3b). An initial burst release was observed within the first 2 h, with ∼42 % of Cur released, followed by a sustained release phase reaching ∼87 % after 8 h. In contrast, the aqueous Cur suspension exhibited a much slower and limited release, with ∼25 % at 2 h and only ∼51 % at 8 h with the similar biphasic release behavior noted with Cur-HES. This smaller burst followed by very slow release observed with Cur suspension may be explained by the limited solubility of Cur in the suspension medium (Abdelbary et al., 2019). These results clearly indicate the superiority of the vesicular system in enhancing curcumin release compared to the suspension. Statistical analysis (One-Way ANOVA) confirmed that the difference in drug release between the Cur-HES formulations and the Curcumin suspension was highly significant at all time points (*p* ˂ 0.001).

The burst phase from Cur-HES may be due to the presence of surface-associated or unentrapped drug, as well as the contribution of hydrophilic components such as Tween 20 and Cremophor EL. These surfactants facilitate rapid hydration of the vesicular surface and enhance the diffusion of unentrapped Cur into the release medium (Fahmy et al., 2025). On the other hand, the subsequent sustained release phase can be explained by the contribution of Span 80 and cholesterol, which provide a stable, lipophilic environment within the vesicular bilayer. These lipophilic domains hinder the rapid diffusion of the entrapped drug, thereby prolonging its release (Ammar et al., 2020). Moreover, the incorporation of HA not only improved the aqueous dispersibility of Cur and enhanced the vesicular stability by reducing aggregation, but also contributed to controlled release through hydrogen bonding interactions with Cur, thereby retarding its diffusion (Li et al., 2020).

When compared with literature, the observed release behavior is in agreement with previous findings on vesicular systems such as terpesomes (Albash et al., 2021), glycerosomes (Younes et al., 2024), and niosomes (Gupta et al., 2011), which similarly exhibited biphasic patterns with an initial burst followed by prolonged release. Importantly, the superior release of curcumin from Cur-HES at physiological pH compared with suspension suggests enhanced solubilization and sustained release, leading to improved intestinal absorption and subsequent hepatic delivery.

Kinetic analysis further supported these observations. The release of Cur from Cur-HES fitted well with the Higuchi diffusion model, indicating a diffusion-controlled mechanism from the vesicular matrix. Conversely, the release of Cur from the aqueous suspension followed a first-order kinetic pattern, where the release rate was mainly dependent on the remaining drug concentration, consistent with its limited solubility (Ahmed et al., 2025a).

Taken together, these results highlight the efficiency of Cur-HES in providing both rapid initial release and prolonged delivery, features that are critical for maintaining therapeutic plasma levels and maximizing the hepatoprotective action of Cur.

#### 3.3.4 Anti-oxidant assay

As illustrated in Fig. 3c, the formulated system demonstrated a remarkable enhancement in antioxidant performance when compared with pure curcumin. The IC₅₀ value notably decreased to 2.59 μg/mL for the optimized HES formulation, indicating an approximately 4.25-fold increase in scavenging activity. This significant improvement suggests that nano-encapsulation within the HES matrix effectively amplifies the intrinsic antioxidant potential of curcumin. The observed enhancement can be ascribed to several formulation-driven mechanisms: (i) improved dispersion and solubility of curcumin in the aqueous phase; (ii) protection against oxidative and enzymatic degradation, preserving molecular integrity; and (iii) increased surface interaction with reactive species due to the nanoscale dimensions and favorable physicochemical properties of the system. Collectively, these factors promote faster and more efficient radical neutralization.

#### 3.3.5 Physical stability study

The optimal Cur-HES formula maintained its physical characteristics after storage at refrigerated conditions for three months, with no noticeable changes in physical appearance. Visual inspection revealed the absence of vesicular aggregation, indicating good physical stability. As shown in Table 5, statistical analysis showed no significant differences (*p* > 0.05) in % EE, PS, PDI, or ZP between freshly prepared and stored formulations, confirming that the nanovesicles retained their structural integrity during storage (Younes et al., 2024).

**Table 5:** Effect of short-term storage on the optimized Cur-HES formula.

Response	Fresh	Stored	P-value^a^
Y_1_: EE %	88.4 ± 0.9	86.3 ± 0.8	0.129
Y_2_: PS (nm)	105.2 ± 1.6	108.3 ± 2.2	0.613
Y3: PDI	0.19 ± 0.02	0.18 ± 0.01	0.123
Y_4_: ZP (mV)	−20.9 ± 1.3	−21.9 ± 2.8	0.713

Abbreviations: EE %, percent entrapment efficiency; Cur, curcumin; PS, particle size; ZP, zeta potential; PDI, polydispersity index; HES, Hyaluronic Acid-Modified Edge-Activated Spanlastics.

The data presented are the mean ± SD (*n* = 3).

a One-way ANOVA analysis to compare the fresh and stored optimum formula.

Moreover, the similarity factor (*f₂*) between the release profiles of fresh and stored formulation was 81.89, reflecting the preservation of release characteristics (Fig. 3b) (Ahmed et al., 2024b). The high stability can be explained by the combined effects of the formulation components. Importantly, the high negative zeta potential induced electrostatic repulsion that prevented vesicular aggregation (Ahmed et al., 2025a), while cholesterol contributed to bilayer stabilization and rigidity (Abdelbari et al., 2021). Additionally, the surface-active properties of Tween 20, Span 80, and Cremophor EL provided steric hindrance, further reducing the possibility of aggregation (Ahmed et al., 2022b; Roy et al., 2020). Moreover, the robust surface charge, primarily contributed by the HA shell, provides the necessary colloidal stability to prevent particle settling and aggregation (Roy et al., 2020).

Furthermore, the nanoscale dimensions of the vesicles also played a key role in their stability. Smaller vesicles exhibit greater Brownian motion, which minimizes sedimentation and aggregation. Their reduced size is typically associated with lower polydispersity and more uniform distribution, both of which enhance dispersion stability. Moreover, the small vesicle size provides a higher surface area, leading to greater exposure of surface charges. This enhances electrostatic repulsion among vesicles and reduces the likelihood of aggregation (Ahmed et al., 2024c; Roy et al., 2021). Collectively, these findings confirm the satisfactory stability of Cur-HES under refrigerated conditions, ensuring consistency of physicochemical and release properties throughout the storage period.

Moreover, the Encapsulation Efficiency (EE%) remained high without significant statistical change, which is a critical indicator of the robust vesicular structure and successful protection against drug leakage over time (Vasam et al., 2023). Collectively, the insignificant changes observed in all evaluated parameters confirm that the formulation components successfully stabilized the nanocarrier system for at least three months under refrigeration.

### 3.4 *In vivo* study of the optimized Cur-HES

#### 3.4.1 Effect of optimized Cur-HES on CCl4- induced liver damage

**Fig. 4:**
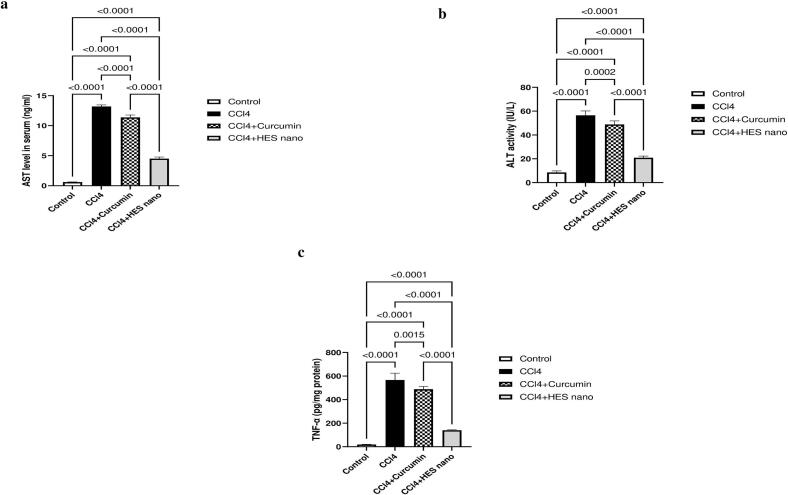
Statistical comparison of liver damage marker enzymes (a) aspartate transaminase (AST) and (b) alanine transaminase (ALT), and Inflammatory marker (c) TNF-α levels in control and experimental animals. (Statistical significance assessed by one-way analysis of variance (ANOVA) followed by Tukey's *post hoc* test (*n* = 6, *p*-values indicating the significant differences between compared groups are stated).

**Fig. 5:**
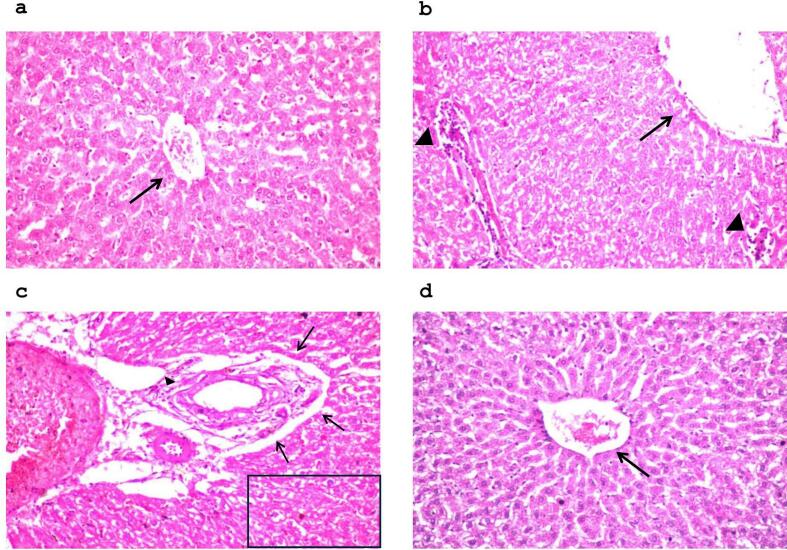
Microscopic photographs (16× magnification) showing (a) normal histological construction of the central vein (black arrow) in control animal, (b) extensive damage where the central vein was dilated (black arrow), in addition to infiltration of inflammatory cells in the portal area in CCl4 model animal (arrow head), (c) dilatation in the portal area with few inflammatory cell infiltration (arrow head), congestion in the portal vein, dilatation of the bile ducts with periductal oedema (black arrow) and hepatocytic degeneration (square) in free curcumin treated animal and (d) preserved hepatocytes with minimal central vein dilatation (black arrow) in optimum Cur-HES formula treated animal.

CCl4 is recognized as a potent hepatotoxic substance which, upon administration, triggers hepatocellular damage. Indeed, hepatocellular injury is detected through investigating hepatic enzymes; Aspartate transaminase (AST) and Alanine transaminase (ALT). AST and ALT are essential for the metabolic function of the liver. Upon hepatocellular injury, AST and ALT are released with high levels in the blood. Noteworthy, ALT is specific biomarker for hepatic toxicity, while AST can be elevated in heart or skeletal muscle injury (Thakur et al., 2024). In the current study, AST content and ALT activity showed an upsurge upon CCl4 administration to reach 22- and 7- folds respectively in relation to normal group (*p* < 0.0001). However, conventional Cur solution showed modest decrease in the previously mentioned parameters by 13.9- (p < 0.0001) and 13.6 % (*p* < 0.001) respectively in relation to CCl4 rats. Controversially, Cur-HES displayed a radical decrease in AST level and ALT activity by 65.9- and 63.1 % respectively as compared to model group (*p* < 0.0001), demonstrating superior hepatoprotective effect. **(**Fig. 4a, b**).** The previously mentioned results align with the histological findings, which recorded normal histological construction of the central vein and nearby hepatocytes in the parenchyma **(**Fig. 5a**)**. On the other side, the model group showed extensive damage where the central vein was dilated, in addition to infiltration of inflammatory cells in the portal area. Moreover, dilatation of portal vein and bile duct, and hepatocellular degeneration were observed as well **(**Fig. 5b**).** Nonetheless, Cur specimens exhibited dilatation as well in the bile ducts, portal vein, periductal edema, and the portal area showed limited infiltration inflammatory cells accompanying with degeneration in the hepatocytes **(**Fig. 5c**).** Contrariwise, Cur-HES preserved the hepatocytes with minimal central vein dilatation **(**Fig. 5d**).** The histopathological examination confirmed the safety of Cur-HES, as liver tissues displayed nearly normal architecture with the absence of necrotic or degenerative changes. These results support the non-toxic nature of the developed formulation.

#### 3.4.2 Effect of optimized Cur-HES on CCl4-induced hepatic inflammation

CCl4 is known to cause hepatic inflammation upon oral administration. This is exhibited by the increase in TNF-α level induced in model group, where the level of TNF-α reached 30 folds relative to control (*p* < 0.0001). Noteworthy, administration of Cur solution slightly amended such elevation, where the TNF-α decreased by only 13.9 % as compared to model group (*p* < 0.01). However, incorporating Cur in HES produced a drastic decrease in hepatic inflammation as TNF-α decreased by 75.2 % compared to insult group (p < 0.0001) signifying enhanced anti-inflammatory response **(**Fig. 4c**).**

The observed reductions in serum ALT, AST, and TNF-α levels support the hepatoprotective potential of Cur-HES, reflecting attenuation of hepatic injury and inflammation. The enhanced hepatoprotective efficacy of the optimized Cur-HES compared to free Cur suspension against CCl₄-induced hepatic injury can be ascribed to the effective entrapment of Cur within flexible spanlastics nanovesicles. Encapsulation enabled Cur to be delivered in a highly dispersed nanoscale form with enlarged surface area, improving solubility, stability, and cellular availability. It was considered that Cur-HES could serve as a potential approach for hepatic targeting. The average vesicular size of the optimized Cur-HES (105.2 ± 1.6 nm as measured by zetasizer and TEM) falls within the favorable range for efficient hepatic uptake by hepatocytes and Kupffer cells, allowing for intracellular accumulation of curcumin at therapeutically relevant levels (Umek et al., 2021; Saher et al., 2023). This explains the observed attenuation of liver damage, inhibition of pro-inflammatory mediators, and overall promotion of hepatocellular protection following oral administration of Cur-HES, compared to free Cur (Ruiz de Porras et al., 2023; Kusumoputro et al., 2023).

The formulation components of optimized Cur-HES play a central role in its performance and safety. **Span 80**, with its unsaturated alkyl chain, provides an optimal packing arrangement that supports bilayer curvature and prevents vesicular aggregation, thus maintaining particle size stability (Ahmed et al., 2022b). The favorable safety and biocompatibility of Span 80 render it appropriate for long-term internal use, as in the case of optimized Cur-HES (Maher et al., 2023). **Tween 20** in Cur-HES is a hydrophilic nonionic surfactant that plays a stabilizing role in vesicular systems. It enhances the dispersibility of vesicles in the gastrointestinal milieu, preventing aggregation and ensuring efficient presentation of Cur at the absorption site. Moreover, Tween 20 contributes to the interaction with mucosal surfaces, which may prolong gastrointestinal residence and improve Cur absorption (Alkufi and Kassab, 2025). **Cremophor EL**, incorporated as an EA, disrupts the packing of the bilayer and imparts deformability, fluidity and flexibility, enabling the vesicles to squeeze through biological membranes without rupture, which is critical for enhanced intestinal permeation and cellular uptake of the encapsulated Cur (Ansari et al., 2022a; Nadim et al., 2025). **Cholesterol**, on the other hand, stabilizes the vesicular bilayer by reducing permeability and preventing premature leakage of Cur, while still maintaining a balance between rigidity and flexibility required for biological performance. So, it preserves structural integrity of the vesicles, ensuring both stability during storage and controlled release *in vivo (*Ansari et al., *2022b**;* Wagdi et al., *2023**)*. Importantly, **HA** in the formulation provides dual benefits: first, it enhances colloidal stability of the vesicles and promotes mucoadhesion in the gastrointestinal tract, thereby prolonging residence time and drug absorption (Cannito et al., 2022); second, HA interacts specifically with CD44 receptors that are overexpressed on hepatocytes during liver inflammation and fibrosis, facilitating active targeting and preferential hepatic accumulation (Li et al., 2020). Moreover, HA contributes indirectly to hepatoprotection due to its partial antioxidant properties, anti-inflammatory activity, and ability to promote tissue repair (Awadalla et al., 2023). These combined attributes suggest that HA decoration synergizes with the intrinsic deformability of spanlastics to maximize curcumin's hepatoprotective action. Overall, the observed therapeutic outcomes can thus be ascribed to the nanoscale size, structural stability, deformability, and HA-mediated hepatic targeting of Cur-HES, which collectively promote Cur delivery to hepatocytes and mitigate hepatic inflammation and liver damage induced by CCl₄.

## 4 Conclusions

In this study, curcumin-loaded hyaluronic acid-enriched spanlastics (Cur-HES) were successfully formulated by the intracellular accumulation method employing a 2^3^ factorial design. The optimum formulation exhibited high entrapment efficiency, nanosized vesicles with spherical morphology, a stable negative zeta potential, and a biphasic release pattern consistent with diffusion-controlled kinetics. Stability studies confirmed that the formulation retained its physicochemical properties for up to three months under refrigerated conditions. Biochemical and histopathological evaluations in rats demonstrated the superior hepatoprotective activity of the optimized Cur-HES compared to curcumin suspension, as evidenced by significant reductions in liver enzyme levels and preservation of normal liver structure. These findings highlight the ability of Cur-HES to overcome curcumin's poor solubility and bioavailability, providing a more effective approach for mitigating CCl₄-induced liver injury. Collectively, the outcomes of this work suggest that Cur-HES is a promising nanocarrier system for enhancing curcumin's hepatoprotective potential and could represent a novel therapeutic strategy against drug-induced liver injury (DILI). Future investigations focusing on large-scale production, extended *in vivo* validation, and clinical translation are warranted to further establish its applicability.

## CRediT authorship contribution statement

**Sadek Ahmed:** Writing – review & editing, Writing – original draft, Supervision, Investigation, Formal analysis, Conceptualization. **Osama Saher:** Writing – review & editing, Resources, Formal analysis. **Rana M. ElBishbishy:** Resources, Investigation, Formal analysis, Conceptualization. **Mennatullah M. Ibrahim:** Writing – review & editing, Writing – original draft, Formal analysis.

## Ethics approval

The protocol for the *in vivo* study was approved by Research Ethics Committee of Faculty of Pharmacy, Cairo University (REC-FOPCU), Egypt (PI 3677), following the US National Institute of Health (NIH) guidelines for the care and use of laboratory animals (NIH Publication No. 85–23, revised 2011). The study was conducted in full compliance with the ARRIVE guidelines, guaranteeing precise reporting and safeguarding animal welfare.

## Funding

The current work has not received any form of funds from any source.

## Declaration of competing interest

The authors declare that they have no known competing financial interests or personal relationships that could have appeared to influence the work reported in this paper.

## Data Availability

The datasets generated during and/or analyzed during the current study are available from the corresponding author on reasonable request.
